# Design and methods for a community-based intervention to reduce sugar-sweetened beverage consumption among youth: H_2_GO! study

**DOI:** 10.1186/s12889-016-3803-5

**Published:** 2016-11-09

**Authors:** Monica L. Wang, Stephenie C. Lemon, Kristian Clausen, Julie Whyte, Milagros C. Rosal

**Affiliations:** 1 Department of Community Health Sciences, Boston University School of Public Health, 801 Massachusetts Avenue, Crosstown Center 4th floor, Boston, MA 02118 USA; 2 UMass Worcester Prevention Research Center, Division of Preventive and Behavioral Medicine, University of Massachusetts Medical School, Worcester, MA USA; 3 The Lewin Group, Falls Church, VA USA

**Keywords:** Methods, Design, Community-based intervention, Sugar-sweetened beverage consumption, Water consumption, Childhood obesitys

## Abstract

**Background:**

Reducing sugar-sweetened beverage (SSB) intake is an important dietary target among underserved children at high risk for obesity and associated morbidities. Community-based approaches to reduce SSB intake are needed. The use of narrative-based approaches (presenting messages within the context of a story) can facilitate connection with target health messages and empower children as behavior change agents within their families. The H_2_GO! program is a community-based behavioral intervention that integrates narrative-based strategies to reduce SSB consumption and promote water intake among school-age youth and parents.

**Methods:**

Guided by the Social Cognitive Theory and the Social Ecological Model, the H_2_GO! intervention consists of 6 weekly sessions that target beverage knowledge, attitudes, and behaviors through youth-produced messages and narratives to reduce SSB intake and encourage water intake and parent–child activities. To reach underserved youth and families, we identified Boys & Girls Clubs (B&GC) (youth-based community centers that serve an ethnically diverse and predominantly low socioeconomic status population) as a community partner and study setting. Participants (children ages 9–12 years and their parents) will be recruited from B&GC sites in Massachusetts, USA. Intervention efficacy will be assessed through a site-randomized trial (*N* = 2 youth-based community sites, pair-matched for size and racial/ethnic composition) with 54 parent–child pairs (*N* = 108) enrolled per site (*N* = 216 total). The comparison site will carry on with usual practice. Child and parental SSB and water consumption (primary outcomes) and parent and child beverage knowledge and attitudes (secondary outcomes) will be measured via self-report surveys. Additional outcomes include children’s anthropometric data, additional dietary behaviors, and physical activity. Measures will be collected at baseline, 2 and 6 months follow-up. With an estimated 20 % dropout rate, the study will have 80 % power to detect a group difference of 3.9 servings of SSBs per week.

**Discussion:**

Community-based approaches hold potential for decreasing SSB consumption among youth and families, particularly among underserved populations who are at greater obesity risk. This article describes the design and methods of a community-based behavioral intervention designed to reduce SSB consumption among youth and parents/caregivers.

**Trial registration:**

ClinicalTrials.gov NCT02890056. Date of Registration: August 31, 2016

## Background

Sugar-sweetened beverages (SSBs) are aggressively marketed to youth [1], comprise the leading source of added sugars to children’s diet [2], and provide little to no nutritional value [3–6]. Overall SSB intake has increased among U.S. youth over the past 10 years [7], averaging at 224 kcal/day (approximately 11 % of total caloric intake) [2]. Several studies have shown that high SSB intake is strongly linked with childhood obesity [2, 8–14]. Differences in children’s SSB intake parallel disparities in childhood obesity; higher rates of obesity and heavy SSB consumption (>500 kcals/day) are prevalent among low socioeconomic status (SES) children compared to high SES children, and among Latino and Black children compared to White children [8, 15, 16].

In contrast to excessive SSB consumption, research indicates that water intake among U.S. children and adolescents has been inadequate over the past decade [17, 18]. Data from the National Health and Nutrition Examination Survey (2009–2012) indicates that more than half (54.4 %) of youth ages 6–19 were not adequately hydrated (defined as urine osmolality ≥ 800 milliosmoles/kg), with nearly a quarter reporting consuming no plain water at all [18]. With over one third of U.S. children currently overweight or obese and at risk for numerous associated health risks, including having a shorter life expectancy than their parents [19], replacing SSBs with water is an important dietary behavior to target among children at elevated obesity risk.

As the majority of SSBs consumed by children take place at home [4, 8], approaches that address parental behaviors related to SSB and water intake (e.g., modeling, purchasing) are needed. Existing childhood obesity interventions that engage children and parents typically include children as passive recipients (e.g., receiving education from an expert) and utilize didactic approaches. These interventions have yielded modest effect estimates and lacked sustainability [20, 21]. Interventions that empower children as behavior change agents within their families have the potential to enhance child behavior change and parental engagement. The use of narrative-based approaches (presenting messages within the context of a story) may facilitate greater connection with target health messages compared to pure didactic instruction [22]. Examples of narrative-based approaches include integration of comic books, telenovelas, and live peer stories within interventions to reinforce targeted health behaviors. Narrative-based interventions have been associated with positive health behavior change among low SES, racial/ethnic minority adults [22–24].

To reach underserved youth and families, we identified local Boys & Girls Clubs (B&GC) of America as a community partner and intervention setting. B&GCs provide affordable after-school programs for ethnically diverse and predominantly lower socioeconomic status populations through nearly 4000 sites across the U.S. [25]. Central to the B&GC mission is partnering with families to promote healthy behaviors. If shown effective, the intervention will eventually be integrated into existing B&GC health programs delivered by trained B&GC staff. No community-based childhood obesity intervention to our knowledge has empowered children as change agents to convey key health behavior messages to parents. The purpose of this manuscript is to describe the design and methods of a community-based behavioral intervention to target SSB and water intake among an ethnically diverse sample of youth and families.

## Methods

### Study aim and design

**Fig. 1:**
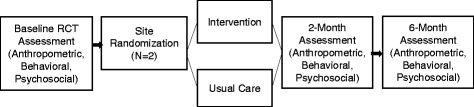
H_2_GO! Study Design

This study aims to assess the feasibility and efficacy of a community-based behavioral intervention (delivered through B&GC sites) targeting SSB and water consumption among 108 parent–child pairs (*N* = 216) through a site-randomized trial. The site-level intervention will take place at two B&GC sites that were pair-matched for size and racial/ethnic composition. One site will be randomized to receive the intervention and the other site will receive usual care. The 6-week intervention consists of weekly group-based sessions with assessments conducted at baseline, 2 and 6 months follow-up (see Fig. 1 for study design). The primary outcomes are child and parental SSB and water intake, assessed via validated survey items. Secondary outcomes include child and parental knowledge and attitudes related to SSBs. Additional outcomes include children’s anthropometrics (height, weight and waist circumference), other dietary behaviors, and physical activity. We hypothesize that child participants in the intervention site will demonstrate reduced SSB intake and increased water intake compared to participants in the comparison site at 2 and 6 months follow-up. Study protocol and procedures were approved by the Boston University Medical Center Institutional Review Board.

### Study setting and population

The B&GC of America is a national organization that provides affordable after-school programs for a large population (~4 million annually) of diverse youth (33 % White, 30 % Black, 23 % Latino) from predominantly low socioeconomic backgrounds through over 4000 club facilities across the U.S. [25]. Given the organization’s commitment to health promotion through health programs delivered by professionals trained in youth development, B&GCs serve as an ideal setting to deliver childhood obesity programs that target ethnically diverse and low SES populations. Given the wide local, state, and national reach of B&GCs to underserved youth, an efficacious behavioral intervention targeting SSB and water intake has high potential for sustainability and dissemination. Two B&GC sites (Worcester and Lowell) in Massachusetts, USA were selected for comparability in youth enrollment size and racial/ethnic composition and geographic spacing (>40 miles apart) to reduce contamination. See Table 1 for participating B&GC site characteristics.

**Table 1:** Characteristics of Boys and Girls Club study sites (2012–2013) in Massachusetts, USA

	Worcester	Lowell
Total youth enrolled	1087	1619
Daily average school year attendance	180	211
% Hispanic	37	33
% Non-Hispanic White	16	20
% Non-Hispanic Black	16	19
% Multiracial	9	10

Parent–child pairs will be recruited from B&GC sites. *Child inclusion criteria* are: ages 9–12 years; current member at the B&GC study site; able to understand and communicate in English, able and willing to provide consent; parental/caregiver permission to participate; and no medical condition that limits ability to change beverage consumption behaviors. *Parental/caregiver inclusion criteria* are: parent/caregiver to a B&GC child member; 18 years or older; able to understand and communicate in English; able and willing to provide consent; and no medical condition that limits ability to change beverage consumption behaviors.

### Intervention condition

#### Development

The intervention was designed to address two behavioral targets: reducing the number of SSB servings consumed per day (recommended guideline of zero SSBs per day) and promoting water consumption (approximately 5–8 cups per day for youth participants and eight cups per day for parental participants). Intervention materials, strategies, format, and content were pilot-tested among a small sample (*N* = 12) of parent–child pairs at a B&GC study pilot site (Lawrence, MA) and finalized based on feedback from parent and child participants, B&GC staff, and observations from the research team. Informed by the Social Cognitive Theory [26] and the Social Ecological Model, the H_2_GO! Intervention was designed to target child and parent participants’ knowledge, attitudes (self-efficacy, outcome expectations, perceived social norms) and behavioral capabilities [27] related to SSB and water consumption.

### Intervention format, content, and strategies

The 6-week intervention consists of group-based weekly sessions (1-h sessions twice a week) delivered by trained B&GC program staff at the B&GC site. Each intervention session consists of a 1-h health module followed by a 1-h narrative module. Topics of the health modules include: understanding the benefits of water, sampling different types of fruit-flavored water, identifying SSBs, exploring the local grocery store, identifying barriers and facilitators to drinking water, and managing triggers for SSBs. See Table 2 for content and objectives targeted during each session. The narrative modules include intervention objectives and activities that reinforce knowledge, attitudes, skills, and behaviors targeted in the previous health component (e.g., if the health topic covers how to identify SSBs, the narrative module guides youth in creating their own messages and narratives through print, audio, or video materials that focus on how to identify SSBs).

**Table 2:** H_2_GO! intervention content

Session	Health component	Narrative component
1	Topic: Water is Good for You! Objectives • Encourage youth to drink water during the session • Discuss benefits of drinking water • Discuss dehydration and over-hydration • Set individual water intake goals • Instruct youth how to keep track of water intake	Topic: Print Narratives to Promote Water Intake Objectives • Encourage youth to recall information learned about water in the previous health session • Guide youth in creating messages (tailored for their parents/caregivers) on drinking water through print narratives (e.g., PSAs, comics)
2	Topic: Re-think Your Drink Objectives • Help youth identify non-sweetened alternative beverages to SSBs • Conduct a group taste test of alternative beverage options • Encourage youth to try different beverage options • Discuss as a group experiences, reactions, and preferences from the taste test	Topic: Print Narratives to Encourage Sampling Different Types of Water Objectives • Encourage youth to recall information and experiences in the previous health session about the different types of water they tasted • Guide youth in creating messages (tailored for their parents/caregivers) on trying different types of water through print narratives (e.g., PSAs, comics)
3	Topic: Find the Facts Objectives • Instruct youth on nutrition labels • Guide youth on experiments with measuring serving size and sugar in a variety of SSBs • Help youth identify SSBs and non-SSBs	Topic: Print Narratives to Explain Beverage Nutrition Labels Objectives • Encourage youth to recall information about nutrition labels, serving sizes, and amount of sugar in SSBs in the previous health session • Guide youth in creating messages (tailored for their parents/caregivers) on reading nutrition labels, serving sizes, and amount of sugar in SSBs through print narratives (e.g., print PSAs, comics)
4	Topic: Explore the Corner Store Objectives • Identify different types of SSBs in a local corner or grocery store by reading labels • Distinguish between SSBs and non-SSBs in a convenience store by reading labels • Go into a store and not purchase SSBs	Topic: Audio Narratives to Explain Different Types of SSBs Objectives • Encourage youth to recall information about different types of SSBs discussed in the previous health session • Guide youth in creating messages (tailored for their parents/caregivers) on the different types of SSBs through audio narratives (e.g., short stories, word skits, raps, songs)
5	Topic: Water, water, everywhere Objectives • Identify opportunities to drink water in various settings • Explain ways to get water in various settings	Topic: Video Narratives to Overcome Barriers to Drinking Water Objectives • Encourage youth to recall information learned about barriers and facilitators to drinking water in the previous health session • Guide youth in creating messages for (tailored for their parents/caregivers) on overcoming barriers to drinking water through group-based video narratives (e.g., script development for skits; rehearsal for filming)
6	Topic: Triggers for SSBs Objectives • Identify triggers for SSBs • Brainstorm ways to avoid SSBs	Topic: Video Narratives to Overcome Barriers to Drinking Water and Manage SSB Triggers Objectives • Encourage youth to recall information learned in health sessions 5 and 6 • Guide youth in creating messages (tailored for their parents/caregivers) on managing SSBs triggers through group-based video narratives (e.g., revise skit scripts as needed; film group skits)
7	Topic: Boys and Girls Club Open House Objectives • Celebrate youth program completion through distribution of certificates and prizes • Strategically display print narratives throughout the club (e.g., by water fountains, vending machines) • Play video and audio narratives created through Club announcement system • Host a free taste test of flavored water for all Boys and Girls Club members	

**Fig. 2:**
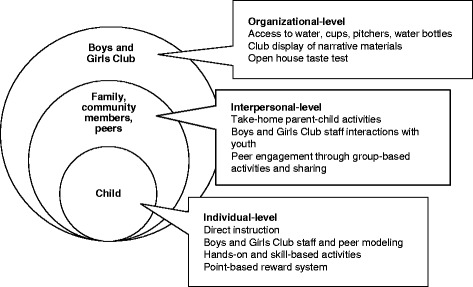
Individual, interpersonal, and environmental-level intervention strategies

Child participants will receive a reusable water bottle and a pictorial intervention booklet. Developed by the study principal investigator (PI) and research assistants, the brightly-colored 45-page booklet was culturally and linguistically-tailored to the study population and included intervention activity worksheets, parent–child take-home activities, fun facts and quizzes, and water and SSB consumption tracking sheets. Activity worksheets will be completed by participants during intervention sessions; and parent–child take-home activities will be completed following each session. Figure 2 presents sample intervention strategies utilized at the individual, interpersonal, and social and physical environmental levels, and Table 3 presents intervention strategies and activities used to target constructs of interest.

**Table 3:** Strategies and constructs targeted by the H2GO! intervention

Intervention strategies and activities	Theoretical constructs targeted			
	Knowledge Related to Sugar-Sweetened Beverages (SSBs) and Water	Attitudes Related to SSB and Water Consumption (outcome expectations, self-efficacy)	Behavioral Capabilities and Skills Related to SSB and Water Consumption (self-monitoring, problem-solving, self-regulation skills)	Behaviors (reducing SSB consumption and promoting water consumption)
Individual level				
*Enactive mastery experiences* (blinded taste tests, local grocery store scavenger hunt of SSBs)	✓	✓	✓	✓
*Modeling* (B&GC staff and peer modeling of drinking water and choosing not to drink SSBs, self-monitoring and goal-setting demonstrations, youth development and sharing of narratives targeting key intervention messages)		✓	✓	✓
*Persuasive communication* (interactive demonstrations on hydration, sugar measurement demonstrations and experiments)	✓	✓		✓
*Active learning* (staff guidance, discussion of experiences, and provision of feedback on: 1) self-monitoring of SSB and water intake; 2) goal-setting on SSB and water intake; 3) problem-solving for barriers to drinking water; and 4) managing SSB triggers)	✓	✓	✓	✓
*Reinforcement* (point-based system to reward attendance and participation, points could be redeemed for prizes; staff provision of verbal encouragement for meeting behavioral targets)		✓		
*Didactic instruction* (instruction on how to read beverage nutrition labels, use of visual aids such as pictorial log sheets and pictorial activities, emphasis on one key message at a time)	✓			✓
Interpersonal level				
*Family support* (parent–child take-home activities, sharing of narratives targeting key intervention messages, parent attendance at Club Open House event)	✓	✓	✓	✓
*Peer modeling* (peer sharing of narrative narratives with guided discussions)		✓	✓	✓
*Group-based guided practice* (Boys and Girls Club staff supervision and provision of group feedback on water and SSB logs, group discussion, group games and activities)	✓	✓	✓	✓
Social and physical environmental level				
*Environmental restructuring* (onsite provision of drinking cups, water pitchers, and reusable water bottles during intervention sessions, display of print, audio, and narrative materials created throughout the Boys and Girls Club)		✓		✓
*Modeling* (Club Open House where youth shared their narratives and invited all Boys and Girls Club members to participate in taste tests)		✓		✓

### Comparison condition

Participants in the comparison site will receive usual care at the B&GC (standard programming) during the study and complete assessments at baseline, 2 and 6 months follow up. The comparison B&GC site will receive an intervention toolkit including intervention materials, protocols, and supplies upon study completion.

### Intervention fidelity

Several strategies will be utilized to maximize intervention fidelity. Prior to study start, the research team will provide in-depth training sessions on all intervention materials and study procedures for B&GC health program staff. The training consists of a comprehensive intervention protocol review, didactic and discussion-based sessions, and mock sessions with feedback provided by research staff. Intervention delivery will be supervised by the PI and 1–2 trained research assistants. Research team members will attend and complete intervention fidelity checks during each intervention session to assess the extent to which intervention activities were completed as designed. Intervention fidelity checks consist of a list of protocol activities for which the extent of fidelity was rated on a scale of 0 to 2 (0 = did not do this activity; 1 = partially completed; 2 = completed), space for comments regarding each activity, and duration of intervention session.

### Participant recruitment

Screening and recruitment will be conducted by the study team (PI, research coordinator) and B&GC staff. The PI will train the research coordinator and B&GC staff in implementing the screening and recruitment protocols and oversee the screening and recruitment process through weekly in-person meetings. In collaboration with B&GC staff at each study site, the PI will design a fact sheet to introduce the study to potential child and parent/caregiver participants. Recruitment packets containing the fact sheet, study eligibility, consent forms, and study procedures will be provided to B&GC staff to distribute among youth within the eligible age range (9–12 years) and parents/caregivers of youth within this age group. B&GC staff will also verbally inform youth of the study, presenting it as a healthy habits program in order to avoid explicit announcement of the underlying hypotheses about SSB consumption and obesity risk.

After an initial pool of interested youth are identified, the research coordinator and B&GC staff will screen youth for eligibility (details provided in Study Setting and Population). The research coordinator will provide further explanation of the study, review study assent forms, and answer questions. The PI will also be available to answer questions and address concerns. Interested and eligible youth will be given study recruitment packets containing study information and consent forms for parents/caregivers. During pick-up time at the B&GC, the research coordinator will meet with parents/caregivers of interested and eligible youth participants, screen parents/caregivers for eligibility (details provided in Study Setting and Population), provide further explanation of the study, review study consent forms, and answer questions. All participants will be informed procedures to protect the confidentiality of data collected and that their care at the B&GC would be in no way affected by study participation. Interested and eligible parent–child pairs will be asked to complete three written consent forms (child assent to participate, parental consent for their child to participate, and parental consent to participate as a study participant). Only parent–child pairs who complete all three forms will be enrolled in the study. Enrolled child and parent/caregiver participants will be asked to complete baseline assessments prior to the intervention start date.

### Study measures

Study assessments will be led by trained researchers and conducted at B&GC sites at baseline and two follow-up time points (2 and 6 months post-baseline).

### Feasibility outcomes

The number of parent–child dyads available for recruitment and retention using a CONSORT diagram [28, 29] and the number and reasons for failure to complete follow-up assessments will be reported. To assess intervention engagement, B&GC staff in the intervention site will track child intervention session attendance rates, number of narrative materials completed per child, percent of parent–child take-home activities completed, and parent and child attendance rates of the family viewing celebration.

Immediately following intervention completion, parent and child participants will rate/report the following indicators on intervention satisfaction and acceptability: overall satisfaction with each intervention component (youth sessions, narrative materials, parent–child take-home activities, and Club open house event); likelihood of referring intervention to a friend; likelihood of participating in a similar intervention targeting a different dietary behavior; intervention aspects perceived to be most useful; and suggestions to improve the intervention. A 5-point Likert scale will be used to assess outcomes 1–4; open ended responses will be used to assess outcomes 5–6.

### Efficacy outcomes

The primary outcomes, child and parent consumption of SSBs and water (frequency, type, and servings of SSBs and water consumed on a typical day in the past 7 days) will be assessed using items from the Youth Risk Behavior Surveillance Survey [30] and a validated youth food-frequency questionnaire (FFQ) [31]. Secondary outcomes (knowledge related to SBBs and water, self-efficacy to reduce SSB intake and drink water) will be assessed among children and parents using modified items from validated surveys [32, 33]. Child height, weight, and waist circumference will be measured at each assessment point using portable digital scales, stadiometers, and tape measures with the participant wearing light clothing and no shoes; measurements will be taken to 2/10^th^ of the nearest inch or pound. Body mass index will be calculated as weight (kg)/height (m^2^). Youth Risk Behavior Surveillance Survey items [30] will be used to assess children’s other dietary behaviors, such as frequency of fruit, vegetable, breakfast, and fast food consumption and intake of non-SSBs (i.e., diet drinks, water, 100 % fruit juice), and children’s physical activity and sedentary behaviors (i.e., frequency and duration of moderate-to-vigorous physical activity and screen time activities). Covariates of interest will be assessed through self-report surveys and include child and parent gender, race/ethnicity, and age; child eligibility for free/reduced priced lunch; and parental education, income, and employment status.

### Power calculation

Our sample size calculations are based on the primary hypothesis that child participants in the intervention site will have reduced SSB intake and increased water intake than child participants in the comparison site at 2 and 6 months follow-up. Results from our pilot data indicate that B&GC children consumed an average of 7.6 servings of SSBs over the past 7 days (SD = 6.5). Assuming that youth in the comparison site will experience no change in SSB intake from baseline to follow-up, a two-sided, two-sample t-test of means with alpha = 0.05 and 80 % power indicates that enrolling 45 children per group will allow us to detect a difference of 3.9 servings of SSBs per week, a clinically meaningful difference of approximately 550–940 kcals per week depending on serving size and SSB type. To account for 20 % dropout, we will enroll 54 parent–child pairs (*N* = 108) per site, yielding a total sample size of 216 participants.

### Analysis plan for feasibility and efficacy outcomes

For feasibility outcomes, recruitment and retention rates will be compared across study sites and t-tests will be used to compare overall rates between the two sites. The number and reasons for failure to complete follow-up assessments will be reported. To analyze intervention engagement, youth session attendance rates for each session, percent of parent–child activities completed per week, number of narrative materials produced per child, and parent and child attendance rates at the club open house event will be reported. Intervention fidelity rates per session will be reported, and reasons for poor fidelity will be explored. For intervention acceptability and satisfaction, we will report descriptive statistics of intervention satisfaction ratings among children and parents and the proportions of children and parents who would recommend the intervention to others.

Distributions, descriptive statistics, and missing values will be examined for all efficacy study variables. Bivariate analyses will compare the characteristics of the intervention and comparison groups using two-tailed chi-square tests for categorical variables and two-tailed t-tests for continuous variables. These results will identify imbalances between groups that will be adjusted for in multivariable analyses. All analyses will utilize an intent-to-treat approach, with each child and parent participant enrolled in the intervention site analyzed as part of the group, regardless of intervention engagement. We will compare mean changes in our primary outcome (children’s SSB intake frequency) and secondary outcomes across intervention conditions using repeated measures mixed models. Generalized estimating equations will be used to account for clustering of observations within sites. Based on the relatively small sample size, complete case analysis with comparisons with baseline data will be used to address missing data as a result of loss to follow up.

## Discussion

Community-based and narrative-based intervention strategies hold potential for decreasing SSB consumption and associated obesity risk among youth and families, particularly among socioeconomically disadvantaged and racial/ethnic minority populations who face disproportionately high obesity rates. Findings from this study will be used to assess efficacy on reducing SSB consumption among youth and families in the USA. If efficacious, the intervention has high potential for dissemination across youth-based settings and will serve as a community-based intervention model to target SSB and water consumption and disparities in childhood obesity.

## Abbreviations

B&GC: Boys and Girls Club
SSB: Sugar-sweetened beverages

## References

[1] Commission UFT (2008). Marketing Food to Children and Adolescents: A Review of Industry Expenditures, Activities, and Self-Regulation.

[2] Reedy J, Krebs-Smith SM (2010). Dietary sources of energy, solid fats, and added sugars among children and adolescents in the United States. J Am Diet Assoc.

[3] Fox S, Meinen A, Pesik M, Landis M, Remington PL (2005). Competitive food initiatives in schools and overweight in children: a review of the evidence. WMJ.

[4] Briefel RR, Wilson A, Gleason PM (2009). Consumption of low-nutrient, energy-dense foods and beverages at school, home, and other locations among school lunch participants and nonparticipants. J Am Diet Assoc.

[5] Gidding SS, Dennison BA, Birch LL, Daniels SR, Gillman MW, Lichtenstein AH, Rattay KT, Steinberger J, Stettler N, Van Horn L (2005). Dietary recommendations for children and adolescents: a guide for practitioners: consensus statement from the American Heart Association. Circulation.

[6] American Academy of Pediatrics CoN (2001). The use and misuse of fruit juice in pediatrics. Pediatrics.

[7] Han E, Powell LM (2013). Consumption patterns of sugar-sweetened beverages in the United States. J Acad Nutr Diet.

[8] Briefel RR, Wilson A, Cabili C, Hedley Dodd A (2013). Reducing calories and added sugars by improving children's beverage choices. J Acad Nutr Diet.

[9] Ludwig DS, Peterson KE, Gortmaker SL (2001). Relation between consumption of sugar-sweetened drinks and childhood obesity: a prospective, observational analysis. Lancet.

[10] Ebbeling CB, Feldman HA, Osganian SK, Chomitz VR, Ellenbogen SJ, Ludwig DS (2006). Effects of decreasing sugar-sweetened beverage consumption on body weight in adolescents: a randomized, controlled pilot study. Pediatrics.

[11] Malik VS, Hu FB (2011). Sugar-sweetened beverages and health: where does the evidence stand?. Am J Clin Nutr.

[12] Malik VS, Popkin BM, Bray GA, Despres JP, Hu FB (2010). Sugar-sweetened beverages, obesity, type 2 diabetes mellitus, and cardiovascular disease risk. Circulation.

[13] Malik VS, Willett WC, Hu FB (2009). Sugar-sweetened beverages and BMI in children and adolescents: reanalyses of a meta-analysis. Am J Clin Nutr.

[14] Hu FB, Malik VS (2010). Sugar-sweetened beverages and risk of obesity and type 2 diabetes: epidemiologic evidence. Physiol Behav.

[15] Harrington S (2008). The role of sugar-sweetened beverage consumption in adolescent obesity: a review of the literature. J Sch Nurs.

[16] Singh GK, Siahpush M, Kogan MD (2010). Rising social inequalities in US childhood obesity, 2003–2007. Ann Epidemiol.

[17] Kant AK, Graubard BI (2010). Contributors of water intake in US children and adolescents: associations with dietary and meal characteristics--National Health and Nutrition Examination Survey 2005–2006. Am J Clin Nutr.

[18] Kenney EL, Long MW, Cradock AL, Gortmaker SL (2015). Prevalence of inadequate hydration among us children and disparities by gender and race/ethnicity: National Health and Nutrition Examination Survey, 2009–2012. Am J Public Health.

[19] Ogden CL, Carroll MD, Kit BK, Flegal KM (2012). Prevalence of obesity and trends in body mass index among US children and adolescents, 1999–2010. JAMA.

[20] Sung-Chan P, Sung YW, Zhao X, Brownson RC. Family-based models for childhood-obesity intervention: a systematic review of randomized controlled trials. Obes Rev. 2012. 10.1111/obr.12000 23136914

[21] Knowlden AP, Sharma M (2012). Systematic review of family and home-based interventions targeting paediatric overweight and obesity. Obes Rev.

[22] Houston TK, Allison JJ, Sussman M, Horn W, Holt CL, Trobaugh J, Salas M, Pisu M, Cuffee YL, Larkin D (2011). Culturally appropriate storytelling to improve blood pressure: a randomized trial. Ann Intern Med.

[23] Rosal MC, Ockene IS, Restrepo A, White MJ, Borg A, Olendzki B, Scavron J, Candib L, Welch G, Reed G (2011). Randomized trial of a literacy-sensitive, culturally tailored diabetes self-management intervention for low-income latinos: latinos en control. Diabetes Care.

[24] Ockene IS, Tellez TL, Rosal MC, Reed GW, Mordes J, Merriam PA, Olendzki BC, Handelman G, Nicolosi R, Ma Y (2012). Outcomes of a Latino community-based intervention for the prevention of diabetes: the Lawrence Latino Diabetes prevention project. Am J Public Health.

[25] Boys and Girls Club of America: Facts and Figures [http://www.bgca.org/whoweare/Pages/FactsFigures.aspx]. Accessed 10 Oct 2013.

[26] Bandura A. Self-efficacy: Toward a unifying theory of behavioral change. Psychol Rev. 1977;84(2):191–215. 10.1037//0033-295x.84.2.191 847061

[27] Bandura A (1997). Self-efficacy. The exercise of control.

[28] Moher D, Hopewell S, Schulz KF, Montori V, Gotzsche PC, Devereaux PJ, Elbourne D, Egger M, Altman DG (2012). CONSORT 2010 explanation and elaboration: updated guidelines for reporting parallel group randomised trials. Int J Surg.

[29] Altman DG, Schulz KF, Moher D, Egger M, Davidoff F, Elbourne D, Gotzsche PC, Lang T (2001). The revised CONSORT statement for reporting randomized trials: explanation and elaboration. Ann Intern Med.

[30] Eaton DK, Kann L, Kinchen S, Shanklin S, Flint KH, Hawkins J, Harris WA, Lowry R, McManus T, Chyen D (2012). Youth risk behavior surveillance - United States, 2011. MMWR Surveill Summ.

[31] Rockett HR, Breitenbach M, Frazier AL, Witschi J, Wolf AM, Field AE, Colditz GA (1997). Validation of a youth/adolescent food frequency questionnaire. Prev Med.

[32] Rivard C, Smith D, McCann SE, Hyland A (2012). Taxing sugar-sweetened beverages: a survey of knowledge, attitudes and behaviours. Public Health Nutr.

[33] Resnicow K, Yaroch AL, Davis A, Wang DT, Carter S, Slaughter L, Coleman D, Baranowski T (2000). GO GIRLS!: results from a nutrition and physical activity program for low-income, overweight African American adolescent females. Health Educ Behav.

